# What Do Medical Students Actually Want from Learning Communities? A Comprehensive Needs Assessment

**DOI:** 10.15694/mep.2020.000008.1

**Published:** 2020-01-09

**Authors:** Karina Renee Clemmons, Sara Ghori Tariq, James Graham, Jason Scott Mizell, Annlee Taylor Glass-Hicks, Purushottam Bhadra Thapa

**Affiliations:** 1 University of Arkansas for Medical Sciences

**Keywords:** learning communities, student needs assessment, medical students

## Abstract

This article was migrated. The article was marked as recommended.

**Purpose:** The demanding nature of medical education has been well-described. Learning Communities (LCs) have been formed in a number of medical schools to address unmet needs such as wellness, social support, and academic/career counseling. However, there is limited information regarding the student perspective in shaping LC goals and activities. This study examined that perspective using a needs assessment survey.

**Methods:** A formal needs assessment survey was completed by 510 medical students. The survey included 16 Likert-scale items and one open response item. Topics focused on student well-being, career planning, meaningful professional relationships, and academic success.

**Results:** As expected, residency success and academic performance were the domains ranked as most important. Of note, the domain of wellness was ranked as less important overall. Results also varied by medical school year and gender.

**Conclusion:** Formal assessment of student needs can serve as a guide to the development of LC programming, hopefully increasing student engagement.

## Introduction

A growing number of medical schools are implementing learning communities (LC) in order to address student needs ( Champaloux and Keeley, 2016; Slavin, Schindler and Chibnall, 2014; Smith *et al.*, 2016). Although the general objective of LCs is to improve the learning environment for student success in medical school, the structure and specific goals of LCs vary widely. Some LCs are within curriculum, including clinical skills teaching and board preparation, while others are extracurricular, including activities related to professional development and social bonding ( Shochet *et al.*, 2019). Even the names used to describe LCs vary, some of which include advisory colleges, academic houses and Learning Communities.

Several articles describe the process of implementing LCs in medical schools ( Fleming *et al.*, 2013; Stewart *et al.*, 2007; Wasson *et al.*, 2016). Faculty typically decide what types of LC activities may be valuable to students. Yet if the purpose of implementing LCs is to enhance student experience, their active input is essential ( Baños, Noah and Harada, 2019; Ferguson *et al.*, 2009; Rosenbaum *et al.*, 2007). Student needs assessments in the form of questionnaires or structured interviews are seen as a valuable resource for collecting student input related to programs and educational experiences. In an emergency medicine interest group curriculum, faculty and student opinions differed in a formal needs assessment ( Lee, Uijtdehaage and Coates, 2011). An extensive search of the existing literature regarding needs assessments for LCs from a student perspective did not yield any further results. There is a need for research on this topic from the student perspective ( Tackett *et al.*, 2011). An important objective of this study was to assess whether student goals for LCs were consistent with those of the faculty. To our knowledge, this is the first reported student needs assessment during the formation of a LC in a medical school setting.

The purpose of this study was to conduct a formal assessment of the students’ perceived needs from the LC. In other words, what do students perceive to be important needs which might be met in a learning community?

## Methods

This study was a cross-sectional survey offirst (M1), second (M2), and third year (M3) class of medical students to ascertain their perceived needs that might be served in a LC program.

***Setting:*** The College of Medicine (COM) at the University of Arkansas for Medical Sciences (UAMS) offers a four-year M.D. degree. The COM, a Liaison Committee on Medical Education (LCME) accredited public institution, is the only M.D.-granting medical school in Arkansas, a mid-sized largely rural Southern state. Our mission is to create physicians who will serve the medical needs of our state. This study was reviewed by the institutional review board and deemed exempt.

***Learning Communities:*** The COM was facing challenges of a more competitive residency process as well as a need for better academic advising and professional mentoring. In an effort to address these issues, the COM implemented a learning communities program in 2016. Each LC consists of 100 students (25 students per class) with 6 faculty LC advisors (2 PhDs and 4 MDs), except our Northwest regional campus house which includes 60 students and two faculty (with 1 PhD and 1 MD).

***Population:*** The population surveyed included M1-M3 students during the spring of 2016. Male/female ratio for the class of 2019 (M1) was 55% male / 45% female, the class of 2018 (M2) was 62% male / 38% female, and the class of 2017 (M3) was 69% male / 31% female. M4 students were not surveyed because they would not be a part of the newly established LC program.

***Instrument*** *:* The 17-item needs assessment was designed with input from representative faculty and administrators who had been involved in professional development related to learning community implementation (See Appendix 1). The questions covered the following five domains: (1) residency success (guidance on preparing for residency, guidance with residency application and guidance with career choice); (2) academic performance (step preparation, better academic performance in courses, access to other faculty in research); (3) wellness (strategies to decrease burn-out, ways to manage personal stress, ways to manage school related stress, and work-life balance); (4) professional relationships (improved academic collaboration with other students, having meaningful relationships with other students, and having meaningful relationships with faculty advisors; and (5) cross-domains (time management skills, help getting organized, & access to opportunities in community service).

The students were asked to rate each item they would like to see achieved through the academic houses as “not important,” “somewhat important,” and “most important.” It also included demographic questions on age, gender, race, and ethnicity. The survey included a space to write in any other expectations students had for the LCs. The needs assessment was distributed via paper following a class session for two of the classes (M1s and M2s) and electronically for the M3s because the students were located at various clinical sites. The survey was anonymous and voluntary.

***Data Analysis:*** Data were analyzed using the SAS v 9.4 (Cary, NC). Proportions of students responding to specific items were calculated. Statistical significance comparing responses between gender and medical school year were estimated using Chi square test of proportions.

## Results/Analysis

A total of 406 students participated in the needs assessment survey giving a response rate of 80%. Of the respondents, 36.7% were M1 students (N=149), 38.9% (N=158) were M2 students, and 24.4% (N=99) were M3 students. Of those who identified their gender, 61.6% (N=231) were males, 38.4% (N=144) were females, and 31 did not identify a gender. Of those who identified their race, 84.2% (N=304) identified themselves as Caucasian, 15.8% (N=57) identified as non-Caucasian, and 45 students did not identify their race.

Overall the domains students ranked as “most important” were residency success and academic performance. The domains that students felt were less important were wellness, professional relationships, and professional development. The sub-domains that students most often selected as being “most important” included: guidance in preparing for residency (86.6%), the residency application process (85.9%), and help in United States Medical Licensing Examinations (USMLE) Step preparation (70.3%). In addition, assistance in achieving better academic performance in courses (59.1%) and meaningful relationships with faculty advisors (54.8%) were the next two areas of importance.

The sub-domains that students selected most often as “not important” were: help getting organized (37.4%), time management skills (34.8%), ways to manage personal stress (34.0%), work-life balance (26.7%), and improved academic collaboration with other students (26.1%) ( Table 1).

**Table 1.** Listing of Needs Reported by Medical Students to Accomplish Through the Academic Houses (N=406)

Needs	Not Important		Somewhat Important		Most Important	
	N	%	N	%	N	%
**Residency Success**						
Guidance on preparing for residency	3	0.7	51	12.6	350	86.6
Guidance with the residency application process	4	1.0	53	13.2	346	85.9
Guidance with career choice	17	4.2	126	31.2	261	64.6
**Academic Performance**						
Step preparation	27	6.7	92	22.9	282	70.3
Better academic performance in courses	25	6.2	139	34.7	237	59.1
Access to other faculty in research	74	18.4	209	52.1	118	29.4
**Wellness**						
Strategies to decrease burn-out	66	16.3	194	47.8	146	36.0
Ways to manage my personal stress	137	34.0	183	45.4	83	20.6
Ways to manage my school related stress	91	22.4	209	51.5	106	26.1
Work-life balance	108	26.7	200	49.4	97	24.0
**Professional Relationships**						
Improved academic collaboration with other students	105	26.1	219	54.5	78	19.4
Having meaningful relationships with other students	69	17.0	183	45.1	154	37.9
Having meaningful relationships with my faculty advisors	23	5.7	160	39.5	222	54.8
**Cross-Domain Needs**						
Time management skills	141	34.8	176	43.5	88	21.7
Help getting organized	152	37.4	180	44.3	74	18.2
Access to opportunities in community service	57	14.2	260	64.7	85	21.1

The three major themes that emerged from the wide range of comments that students wrote about their expectations from the LCs included help with the residency process and match, relationship with faculty, and relationship with other students.

*Perceived Needs by Medical School Class.* Of the five sub-domains most often selected as “most important,” the perceived needs varied by medical school class for guidance with the residency application process (p=.004), USMLE Step preparation (p<.0001), better academic performance in courses (p=.004), and having meaningful relationships with faculty advisors (p=.004). Fewer M3 students selected these sub-domains as “most important” compared to M1 and M2 students. The sub-domain of guidance in preparing for residency did not vary by class ( Table 2).

**Table 2.** Listing of Needs Reported by Medical Students to Accomplish Through the Academic Houses (N=406) by Medical School Year

Needs	Class	Not Important		Somewhat Important		Most Important		X ^2^	P Value
		N	%	N	%	N	%		
**Residency Success**									
Guidance on preparing for residency	M1	1	0.7	20	13.4	128	85.9	-	0.0003 *
	M2	0	0.00	15	9.6	142	90.5		
	M3	2	2.0	16	16.3	80	81.6		
Guidance with the residency application process	M1	0	0.0	18	10.7	133	89.3	-	<.0001 *
	M2	1	0.6	16	10.1	141	89.2		
	M3	3	3.1	21	21.9	72	75.0		
Guidance with career choice	M1	7	4.7	54	36.5	87	58.8	16.7	0.002
	M2	2	4.7	37	23.4	119	75.3		
	M3	8	8.2	35	35.7	55	56.1		
**Academic Performance**									
Step preparation	M1	0	0.0	14	9.4	135	90.6	52.7	<.0001
	M2	14	8.9	45	28.7	98	62.4		
	M3	13	11.7	33	34.7	49	51.6		
Better academic performance in courses	M1	7	4.7	39	26.4	102	68.9	15.4	0.004
	M2	9	5.8	54	34.8	92	59.4		
	M3	9	9.2	46	46.9	43	43.9		
Access to other faculty in research	M1	28	19.1	85	57.8	34	23.1	7.1	0.13
	M2	24	15.2	79	50.0	55	34.8		
	M3	22	22.9	45	46.9	29	30.2		
**Wellness**									
Strategies to decrease burn-out	M1	19	12.8	78	52.4	52	34.9	4.9	0.302
	M2	27	17.1	68	43.0	63	39.9		
	M3	20	20.2	48	48.5	31	31.3		
Ways to manage my personal stress	M1	47	31.5	75	50.3	27	18.1	3.2	0.52
	M2	53	33.5	68	43.0	37	23.4		
	M3	37	38.5	40	41.7	19	19.8		
Ways to manage my school related stress	M1	34	22.8	83	55.7	32	21.5	2.8	0.59
	M2	35	22.2	78	49.4	45	28.5		
	M3	22	22.2	48	48.5	29	29.3		
Work-life balance	M1	51	34.2	71	47.7	27	18.1	13.1	0.01
	M2	32	20.4	75	47.8	50	31.9		
	M3	25	25.3	54	54.6	20	20.2		
**Professional Relationships**									
Improved academic collaboration with other students	M1	32	21.6	88	59.5	28	18.9	5.4	0.24
	M2	41	26.1	81	51.6	35	22.3		
	M3	32	33.0	50	51.6	15	15.5		
Having meaningful relationships with other students	M1	21	14.1	64	43.0	64	43.0	13.9	0.008
	M2	23	14.6	68	43.0	67	42.4		
	M3	25	25.3	51	51.5	23	23.2		
Having meaningful relationships with my faculty advisors	M1	7	4.7	63	42.3	79	53.0	15.1	0.004
	M2	6	3.8	50	31.7	102	64.6		
	M3	10	10.2	47	48.0	41	41.8		
**Cross-Domain Needs**									
Time management skills	M1	58	38.9	64	43.0	27	18.1	5.6	0.23
	M2	48	30.6	66	42.0	43	27.4		
	M3	35	35.4	46	46.5	18	18.2		
Help getting organized	M1	59	39.6	70	47.0	20	13.4	10.4	0.03
	M2	54	34.2	63	39.9	41	26.0		
	M3	39	39.4	47	47.5	13	13.1		
Access to opportunities in community service	M1	20	13.4	96	64.4	33	22.2	0.5	0.97
	M2	22	13.9	102	64.6	34	21.5		
	M3	15	15.8	62	65.3	18	19.0		

* Fisher’s Exact Test

Of the sub-domains that students selected most often as “not important,” the perceived needs varied by medical school class for work-life balance (p=.01) and help getting organized (p=.03). More M1 students selected these as “not important” compared to M2 and M3 students. The sub-domains of time management skills, ways to manage personal stress, and improved academic collaboration with other students did not vary by class ( Table 2).

*Perceived Needs by Gender.* Of the sub-domains that students most often selected as being “most important,” only two varied by gender: having meaningful relationships with other students (p=.007) and meaningful relationships with faculty advisors (p<.0001). Female students more often selected these as being “most important” compared to male students. The other three sub-domains of guidance for preparing for residency, Step preparation, and better academic performance in courses did not vary by gender ( Table 3).

**Table 3.** Listing of Needs Reported by Medical Students to Accomplish Through the Academic Houses (N=406) by Gender *

Needs	Gender	Not Important		Somewhat Important		Most Important		X ^2^	P Value
		N	%	N	%	N	%		
**Residency Success**									
Guidance on preparing for residency	Male	2	0.9	33	14.4	194	84.7	-	0.025 **
	Female	0	0.0	15	10.4	129	34.6		
Guidance with the residency application process	Male	4	1.7	35	15.2	191	83.0	-	0.002 **
	Female	0	0.0	12	8.3	132	91.7		
Guidance with career choice	Male	10	4.4	81	35.2	139	60.4	5.5	0.065
	Female	5	3.5	35	24.3	104	72.2		
**Academic Performance**									
Step preparation	Male	17	7.5	48	21.1	163	71.5	1.4	0.5
	Female	7	4.9	35	24.5	101	70.6		
Better academic performance in courses	Male	16	7.0	83	36.2	130	56.8	2.8	0.24
	Female	6	4.2	44	31.0	92	64.8		
Access to other faculty in research	Male	49	21.4	113	49.3	67	29.3	3.1	0.21
	Female	20	14.1	78	54.9	44	31.0		
**Wellness**									
Strategies to decrease burn-out	Male	44	19.1	106	45.9	81	35.1	5.0	0.08
	Female	15	10.4	74	51.4	55	38.2		
Ways to manage my personal stress	Male	84	36.5	102	44.4	44	19.1	2.5	0.28
	Female	41	28.7	73	51.1	29	20.3		
Ways to manage my school related stress	Male	58	25.1	119	51.5	54	23.4	4.6	0.10
	Female	23	16.0	80	55.6	41	28.5		
Work-life balance	Male	75	32.6	101	43.9	54	23.5	8.6	0.01
	Female	28	19.4	82	56.9	34	23.6		
**Professional Relationships**									
Improved academic collaboration with other students	Male	60	26.3	129	56.6	39	17.1	2.6	0.27
	Female	32	22.2	78	54.2	34	23.6		
Having meaningful relationships with other students	Male	48	20.8	103	44.6	80	34.6	9.8	0.007
	Female	13	9.0	67	45.6	64	44.4		
							
Having meaningful relationships with my faculty advisors	Male	16	6.9	104	45.0	111	48.1	18.6	<.0001
	Female	3	2.1	40	27.8	101	70.1		
**Cross-Domain Needs**									
Time management skills	Male	92	40.0	93	40.4	45	19.6	5.3	0.07
	Female	41	28.5	67	46.5	36	25.0		
Help getting organized	Male	94	40.7	100	43.3	37	16.0	4.5	0.10
	Female	45	31.3	66	45.8	33	22.9		
Access to opportunities in community service	Male	35	15.4	156	68.4	37	16.2	10.0	0.007
	Female	16	11.1	85	59.0	43	29.9		

* Missing gender values, n=35

* Fisher’s Exact Test

Of the sub-domains that students most often selected as “not important,” only work-life balance varied by gender, where more males reported it as being not important (p=.01). The other four sub-domains of help getting organized, time management skills, ways to manage personal stress, and improved academic collaboration with other students did not vary by gender ( Table 3).

## Discussion

To the best of our knowledge, this is the first published survey of medical students’ expectations and needs regarding their participation in a learning community. The focus of LCs in many medical schools includes promoting wellness, developing leadership skills, improving interpersonal relationships, and creating a more positive learning environment ( Fleming *et al.*, 2013; Stewart *et al*., 2007). We anticipated that our students would have similar priorities. The survey results in some domains were expected, while other results were not. Although there was substantial overlap in faculty and student opinions of what students could gain from participation in LCs, there were also areas where student priorities were different from faculty priorities.

As expected, domains related to career success such as academic performance and residency success were high priority for students across years. While meaningful relationships with faculty advisors and other students were also high priority for the students across years, they were significantly higher priority for female students. The focus on career success was not surprising given the increased challenges surrounding board exams, residency selection, and the match process. In addition, interests and needs varied according to program year. For example, M1s and M2s were more interested in USMLE Step 1 preparation, whereas M3s were more interested in the residency selection process. The planning processes for establishing the LCs incorporated these expected findings.

One unexpected finding was that although the M3 students noted that residency preparation was their highest priority, the proportion who ranked that as most important was lower than the M1s and M2s. This may be the result of the fact that the M3s were already connected with specialty advisors and were receiving mentoring, albeit outside of the LC. In addition, the college holds required meetings where residency selection and National Residency Match Program (NRMP) preparation is covered throughout the year.

Another unexpected finding was in the wellness domain. Faculty felt work-life balance and stress management was an important need for students and prioritized this as one of the main objectives of the LCs. However, students reported wellness as a lower priority when compared with academics. A possible explanation for this is that students view personal wellness as something they should self-manage and distinct from their school work. Thus, they view wellness as less of a priority when competing with the demands of medical school, impending USMLE Step examinations, and residency selection process. Nevertheless, approximately one-third of the students ranked the four wellness sub-domains as “most important.” Given the well-documented high rates of depression and stress among medical students in the literature ( Wasson *et al*., 2016; Dyrbye, Thomas and Shanafelt, 2006), it is critical to educate students in the importance of wellness and proactively incorporate such activities into the LCs.

The results of this unique needs assessment survey provided valuable information that were used to inform development and modification of our LC activities. More proactive wellness programs were offered to students. The COM started an annual Academic House Olympics for the LCs, where students bring family and friends to participate in friendly, competitive, recreational activities. Longitudinal 1:1 faculty-student mentoring across all four years was established as part of the LC mentoring program. Community service was incentivized within the LC structure. In addition, the feedback also provided ideas to implement in the medical school curriculum. For example, a required longitudinal senior residency preparation course was added to the curriculum. USMLE Step 1-style question review sessions were also added to the pre-clinical curriculum.

A limitation of this study is that this needs assessment was a one-time cross-sectional survey. It is possible that perceived needs may change over time. Nevertheless, the findings of this study provide valuable insight to schools who want to begin or improve their LC programs. This needs assessment model will be used to make future improvements to our LCs.

## Conclusion

Several findings of the study would be helpful for other schools as they plan the development or improvement of LCs. Activities that promote academic success and residency preparation, which students ranked as highest priorities, are likely to be well accepted. However, students may be less likely to accept LC activities designed to address wellness, time management, and help with organization because they may not perceive them as high priorities. It should nevertheless be considered a critical mission of an LC to educate students as to why these areas are important and integral to their academic and career success, and to provide students with skills to improve in those areas.

## Take Home Messages

• Formally assessing student needs is important in programatic and curriculum planning.
• Students ranked academic success and residency preparation as highest priorities for learning community programming.
• Students may be less likely to accept learning community activities designed to address wellness, time management, and help with organization because they may not perceive them as high priorities.

## Notes On Contributors

Karina Renee Clemmons, EdD, is an Associate Professor at the University of Arkansas for Medical Sciences College of Medicine.

Sara Ghori Tariq, MD, is a Professor at the University of Arkansas for Medical Sciences College of Medicine.

James Graham, MD, is a Professor at the University of Arkansas for Medical Sciences College of Medicine.

Jason Scott Mizell, MD, is an Associate Professor at the University of Arkansas for Medical Sciences College of Medicine.

Annlee Taylor Glass-Hicks, BS, is a medical student at the University of Arkansas for Medical Sciences College of Medicine.

Purushottam Bhadra Thapa, MD, is a Professor at the University of Arkansas for Medical Sciences College of Medicine.

## Appendices

### Appendix 1 Learning Community Student Needs Assessment

Please complete the following information.

Age: _______________________

Gender: ____________________

Race/ethnicity: _______________

**Table:**

Which of the following do you WANT to achieve from involvement in the Learning Community/Academic House?Rate on a scale of 1-3 from Not important to Most important.	Not Important	Somewhat Important	Most Important
Step Preparation	O	O	O
Access to other faculty in research	O	O	O
Access to opportunities in community service	O	O	O
Better academic performance in courses	O	O	O
Guidance on preparing for residency	O	O	O
Guidance with the residency application process	O	O	O
Guidance with career choice	O	O	O
Improved academic collaboration with other students	O	O	O
Having meaningful relationships with other students	O	O	O
Having meaningful relationships with my faculty advisors	O	O	O
Ways to manage my school-related stress	O	O	O
Way to manage my personal stress	O	O	O
Strategies to decrease burn-out	O	O	O
Time Management Skills	O	O	O
Help getting organized	O	O	O
Work-life balance	O	O	O

Other areas you would like to gain from the Learning Community/Academic House:
