# Supplementary material for: What Do Medical Students Actually Want from Learning Communities? A Comprehensive Needs Assessment
Source: MedEdPublish (2016). 2020 Jan 9;9:8. [Version 1] doi: 10.15694/mep.2020.000008.1 (PMC10697527; doi:10.15694/mep.2020.000008.1)
Supplement: Peer review history

# Reviewer report: Reviewer response for version 1

**DOI:** 10.21956/mep.19961.r30328

**Published:** 2020-04-23

**Authors:** Ken Masters

**Affiliations:** 1 Sultan Qaboos University

This review has been migrated. The reviewer awarded 4 stars out of 5 An interesting paper on what medical students actually want from learning communities. This is an important topic, as my experience has frequently been that faculty direct the creation of the LC based upon perceived needs, and are guided strongly by the curriculum and teaching circumstances and their own experiences. In this case, the authors have approached the problem from the other side: identifying student needs. These may not always coincide with curriculum and professional requirements, but is a useful approach to gathering information on which to build.The study has been well conducted, has clear goals, and has mostly met those goals. I do feel, however, that given the wide disparity between the needs expressed by the students, and massive impact of burnout and other issues that is known to hit residents, that the study results would have been strengthened with follow-up focus groups, because one area that would need to be explored more detail is what to do with the information. I would venture that it would be a grave disservice to the students if the school decided to tailor their effort only at these needs. One interpretation of the results may be that the students may have no idea about what they are about to face. On the other hand, another interpretation may be that the current student support system is so well structured, and students are already receiving such high-quality support, that they feel they have the confidence to concentrate on other issues. Before the school restructures any efforts, it would be useful to round off this research by examining those aspects in more detail.Nevertheless, a useful read to gauge the students’ perspectives on what is required.

Reviewer Expertise:

NA

No decision status is available

# Reviewer report: Reviewer response for version 1

**DOI:** 10.21956/mep.19961.r30329

**Published:** 2020-02-12

**Authors:** Trevor Gibbs

**Affiliations:** 1 AMEE

This review has been migrated. The reviewer awarded 4 stars out of 5 An interesting paper that I enjoyed reading. I believe it is an important paper during a time when student / doctor resilience is being discussed, as is the burn-out and stress levels of students. One of the main reasons for student stress is often quoted as an early need to be looking toward the future and enabling a clinical position, which I feel supports your conclusions.As you say, the paper does have its limitations but despite those I do think that the paper deserves recognition and I would recommend it to all curriculum planners

Reviewer Expertise:

NA

No decision status is available
